# Supplementary material for: Mutant p53 gains oncogenic functions through a chromosomal instability-induced cytosolic DNA response
Source: Nat Commun. 2024 Jan 2;15:180. doi: 10.1038/s41467-023-44239-2 (PMC10761733; doi:10.1038/s41467-023-44239-2)
Supplement: Supplementary file 1 — Supplementary information [file 41467_2023_44239_MOESM1_ESM.pdf]

Supplementary Information for

Mutant p53 gains oncogenic functions through a chromosomal instability-induced cytosolic DNA response

Mei Zhao, Tianxiao Wang, Frederico O. Gleber-Netto, Zhen Chen, Daniel J. McGrail, Javier A. Gomez, Wutong Ju, Mayur A. Gadhikar, Wencai Ma, Li Shen, Qi Wang, Ximing Tang, Sen Pathak, Maria Gabriela Raso, Jared K. Burks, Shiaw-Yih Lin, Jing Wang, Asha S. Multani, Curtis R. Pickering, Junjie Chen, Jeffrey N. Myers & Ge Zhou

Corresponding authors: Jeffrey N. Myers, jmyers@mdanderson.org; Ge Zhou, gzhou@mdanderson.org

The PDF file includes:

Supplementary Figs. 1-8
Figure legends to Supplementary Figs. 1-8

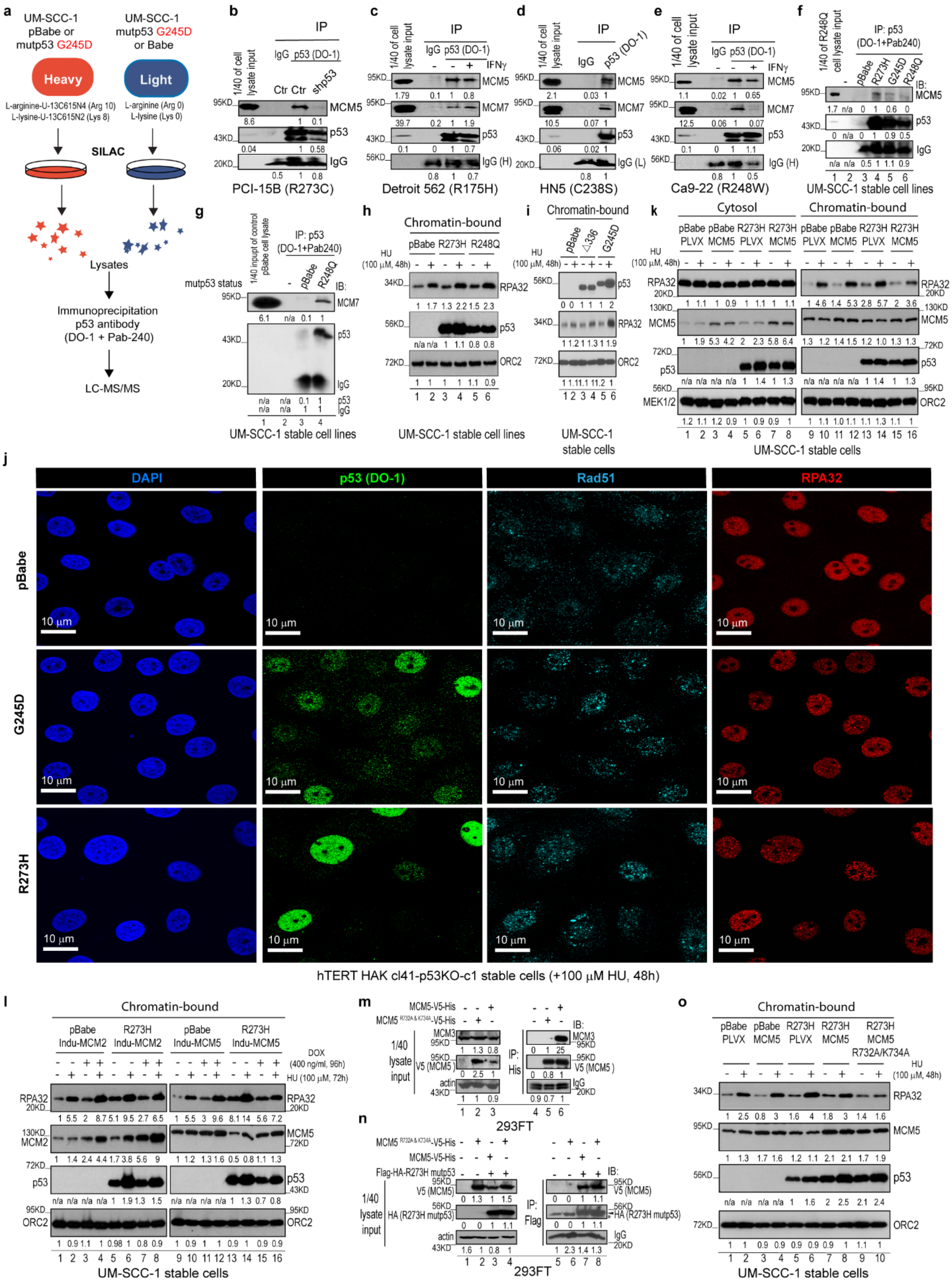

a
UM-SCC-1 pBabe or mutp53 G245D
UM-SCC-1 mutp53 G245D or Babe
Heavy
Light
L-arginine-U-13C615N4 (Arg 10) L-lysine-U-13C615N2 (Lys 8)
L-arginine (Arg 0) L-lysine (Lys 0)
SILAC
Lysates
Immunoprecipitation p53 antibody (DO-1 + Pab-240)
LC-MS/MS
b
IP
IgG p53 (DO-1)
1/40 of cell lysate input
Ctr Ctr shp53
MCM5
p53
IgG
PCI-15B (R273C)
c
1/40 of cell lysate input
IgG p53 (DO-1)
IFNγ
MCM5
MCM7
p53
IgG (H)
Detroit 562 (R175H)
d
HN5 (C238S)
e
Ca9-22 (R248W)
f
IP: p53 (DO-1+Pab240)
1/40 of R248Q cell lysate input
pBabe R273H G245D R248Q
UM-SCC-1 stable cell lines
g
mutp53 status
1/40 input of control pBabe cell lysate
MCM7
UM-SCC-1 stable cell lines
h
Chromatin-bound
pBabe R273H R248Q
HU (100 μM, 48h)
RPA32
p53
ORC2
UM-SCC-1 stable cell lines
i
Chromatin-bound
pBabe △336 G245D
UM-SCC-1 stable cells
k
Cytosol
Chromatin-bound
pBabe PLVX
pBabe MCM5
R273H PLVX
R273H MCM5
MEK1/2
UM-SCC-1 stable cells
j
DAPI
p53 (DO-1)
Rad51
RPA32
pBabe
G245D
R273H
10 μm
hTERT HAK cl41-p53KO-c1 stable cells (+100 μM HU, 48h)
l
Chromatin-bound
pBabe Indu-MCM2
R273H Indu-MCM2
pBabe Indu-MCM5
R273H Indu-MCM5
DOX (400 ng/ml, 96h)
HU (100 μM, 72h)
MCM2
UM-SCC-1 stable cells
m
MCM5-V5-His
MCM5 R732A & K734A-V5-His
1/40 lysate input
IP: His
MCM3
V5 (MCM5)
actin
293FT
n
Flag-HA-R273H mutp53
HA (R273H mutp53)
IP: Flag
293FT
o
Chromatin-bound
R273H MCM5 R732A/K734A
UM-SCC-1 stable cells

Supplementary Fig. 1: mutp53 interacts with MCMs and predisposes cells to replication stress

a, Diagram of SILAC/IP purification of G245D mutp53 interactome in UM-SCC-1 stable cells. LC-MS/MS, liquid chromatography with tandem mass spectrometry. **b**-**e**, p53 antibody (DO-1) IP/Western blot analyses using cell lines with the indicated endogenous mutp53s. shp53, cells with mutp53 knockdown. IFN-γ, 200 IU, 30 min. **f** and **g,** p53 antibody (DO-1 & Pab240) IP/Western blot analyses of UM-SCC-1 stable cell lines with the indicated mutp53s. **h** and **i,** Western blot analyses of chromatin-bound fractions from indicated UM-SCC-1 stable cell lines in response to HU treatment. **j,** IF staining of hTERT HAK cl41-p53KO-c1 stable cells 48 h after treatment with 100 μM of hydroxyurea (HU). Note that more nuclear RPA32 foci were seen in cells expressing mutp53s than in the control cells, which showed homogenous RPA32 staining. **k,** Western blot analysis of cytosol-soluble and chromatin-bound fractions from different UM-SCC-1 stable cell lines in response to HU treatment. **l,** Western blot analysis of chromatin-bound fraction from indicated UM-SCC-1 stable cell lines with doxycycline-inducible expression of MCM2 (indu-MCM2) or MCM5 (indu-MCM5). **m,** His antibody IP/Western blot analysis of HEK293-FT cells co-transfected with V5-His tagged MCM5 or V5-His tagged R732A/K734A mutant MCM5. **n,** Flag antibody IP/Western blot analysis of HEK293-FT cells co-transfected with Flag-HA-tagged R273H mutp53 and V5-His tagged MCM5 or with Flag-HA-tagged R273H mutp53 and V5-His tagged R732A/K734A mutant MCM5. *, non-specific. **o,** Western blot analysis of chromatin-bound fractions from different UM-SCC-1 stable cell lines in response to HU treatment. Source data are provided as a Source Data file.

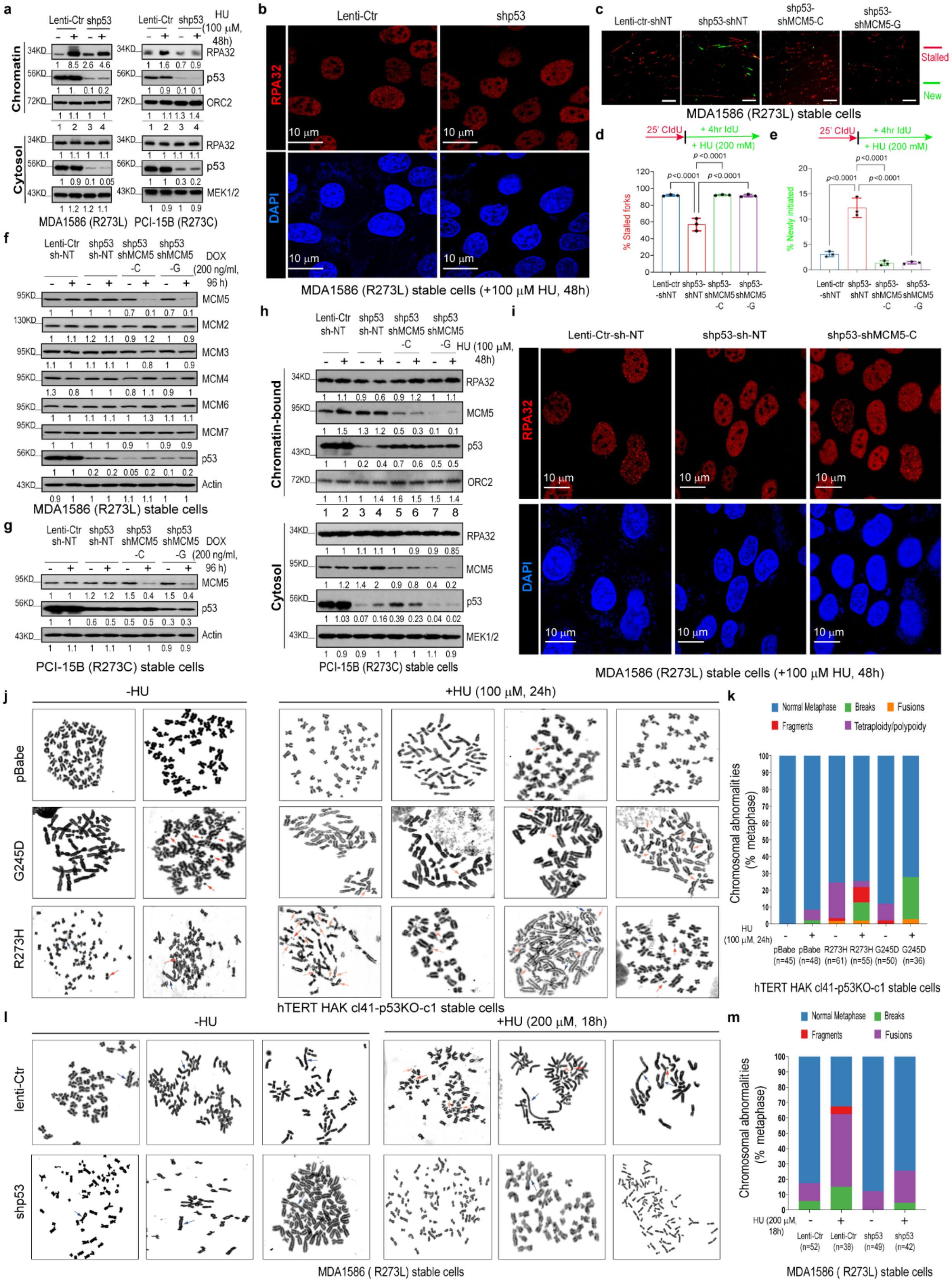

a
Lenti-Ctr shp53 Lenti-Ctr shp53 HU (100 μM, 48h)
Chromatin
Cytosol
RPA32
p53
ORC2
MEK1/2
MDA1586 (R273L) PCI-15B (R273C)
b
Lenti-Ctr
shp53
RPA32
DAPI
10 μm
MDA1586 (R273L) stable cells (+100 μM HU, 48h)
c
Lenti-ctr-shNT
shp53-shNT
shp53-shMCM5-C
shp53-shMCM5-G
Stalled
New
MDA1586 (R273L) stable cells
d
25' CldU
+ 4hr IdU
+ HU (200 mM)
% Stalled forks
p<0.0001
e
% Newly initiated
f
Lenti-Ctr sh-NT
shp53 sh-NT
shp53 shMCM5 -C
shp53 shMCM5 -G
DOX (200 ng/ml, 96 h)
MCM5
MCM2
MCM3
MCM4
MCM6
MCM7
p53
Actin
MDA1586 (R273L) stable cells
g
PCI-15B (R273C) stable cells
h
Chromatin-bound
Cytosol
RPA32
MCM5
p53
ORC2
MEK1/2
PCI-15B (R273C) stable cells
i
Lenti-Ctr-sh-NT
shp53-sh-NT
shp53-shMCM5-C
MDA1586 (R273L) stable cells (+100 μM HU, 48h)
j
-HU
+HU (100 μM, 24h)
pBabe
G245D
R273H
hTERT HAK cl41-p53KO-c1 stable cells
k
Normal Metaphase
Breaks
Fusions
Fragments
Tetraploidy/polypoidy
Chromosomal abnormalities (% metaphase)
HU (100 μM, 24h)
pBabe (n=45)
pBabe (n=48)
R273H (n=61)
R273H (n=55)
G245D (n=50)
G245D (n=36)
hTERT HAK cl41-p53KO-c1 stable cells
l
-HU
+HU (200 μM, 18h)
lenti-Ctr
shp53
MDA1586 (R273L) stable cells
m
Normal Metaphase
Breaks
Fragments
Fusions
Chromosomal abnormalities (% metaphase)
HU (200 μM, 18h)
Lenti-Ctr (n=52)
Lenti-Ctr (n=38)
shp53 (n=49)
shp53 (n=42)
MDA1586 (R273L) stable cells

Supplementary Fig. 2: mutp53 predisposes cells to replication stress and CIN *via* MCM5

a, Western blot analyses of chromatin-bound and cytosol-soluble fractions from the indicated stable cell lines. **b**, IF staining of MDA1586 stable cells in response to HU treatment. Note that parental cells formed more RPA32 foci than did the mutp53 knockdown (shp53) cells. **c**, Representative images of DNA fiber assay of MDA1586 stable cell lines in the presence of HU treatment (200 μM, 4h). Scale bar, 10 μm. **d** and **e**, Summary of DNA fiber assay of MDA1586 stable cell lines in (**c**). error bars represent SD; significances were tested by one-way ANOVA summary with Dunn's multiple comparisons test. **f** and **g**, Western blot analyses of whole cell extracts from stable cells with doxycycline (DOX)-inducible knockdown of MCM5 as indicated. **h**, Western blot analysis using the cytosol and chromatin-bound fractions from PCI-15B stable cell lines treated with HU as indicated. **i**, IF staining of MDA1586 stable cell lines in the presence of HU as indicated. Note that although mutp53 knockdown (shp53-shNT) cells exhibited reduced RPA32 foci formation compared to the control cells (Lenti-Ctr-shNT), further MCM5 knockdown (shp53-shMCM5-C) rescued foci formation. Cells were cultured in medium with DOX (200 ng/mL) for at least 96 h before further HU treatment (**h**,**i**). **j**, Examples of the metaphase spreads of hTERT HAK cl41-p53KO-c1 stable cell lines in the absence or presence of HU. **k**, Summary of chromosomal abnormalities in the metaphase spreads of hTERT HAK cl41-p53KO-c1 stable cell lines in the absence or presence of HU treatment. **l**, Examples of the metaphase spreads of MDA1586 stable cell lines in the absence or presence of HU treatment. **m**, Summary of chromosomal abnormalities in the metaphase spreads of MDA1586 stable cell lines in the absence or presence of HU treatment. Representative chromosomal abnormalities are marked by arrows (pink, breaks; red, fragments; light blue, fusions) (**j**) and (**l**). Source data are provided as a Source Data file.

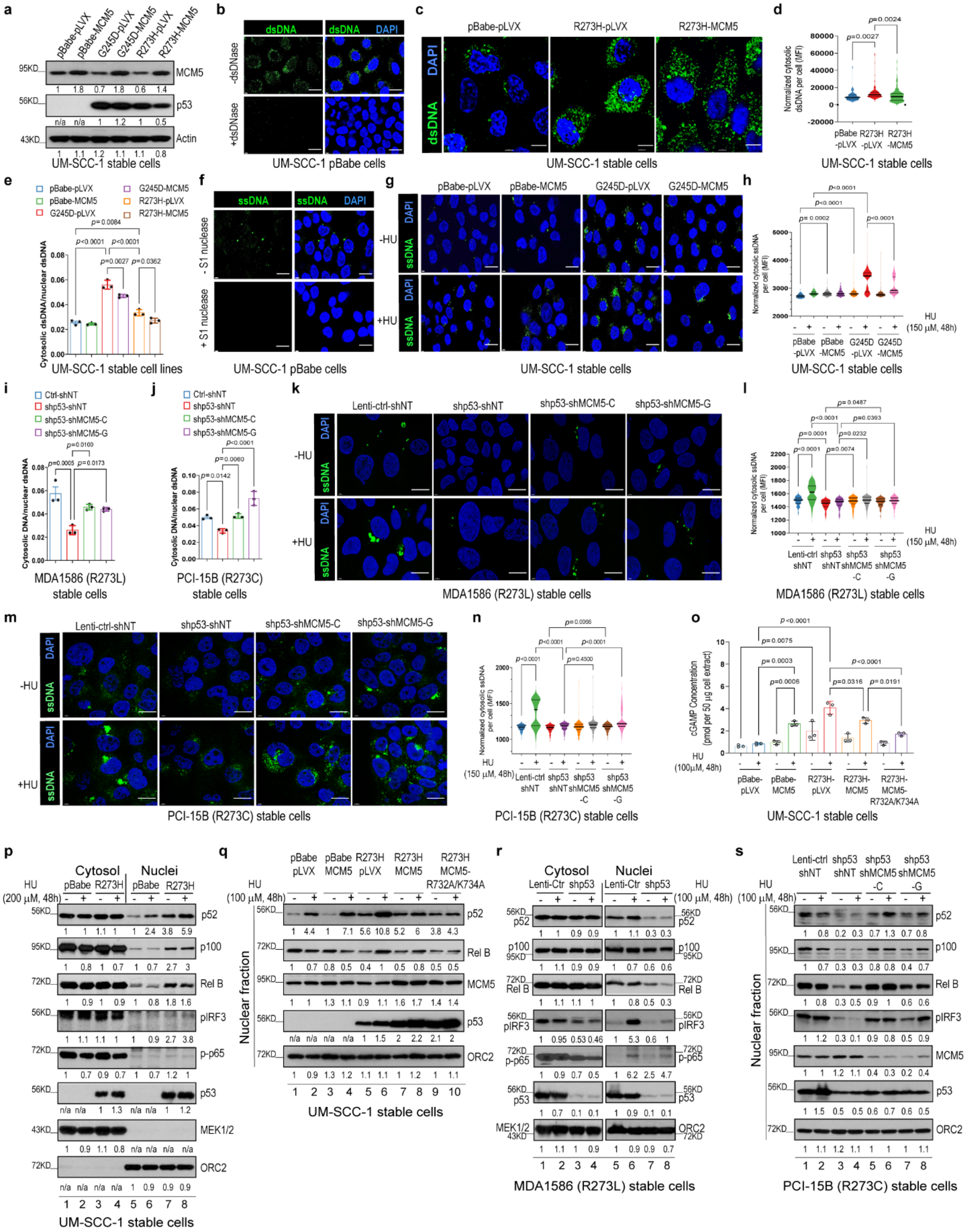

Supplementary Fig. 3: GOF mutp53-MCM5-mediated replication stress and CIN stimulate cytosolic DNA accumulation and NC-NF-κB activation

a, Western blot analysis of whole cell extracts of UM-SCC-1 stable cell lines. **b**, Representative IF staining images of cytosolic dsDNA in UM-SCC-1 stable cells with or without dsDNase. Note that dsDNA staining was eliminated by dsDNase treatment. **c**, Representative IF staining images of cytosolic dsDNA in the indicated UM-SCC-1 stable cells. **d**, Violin plot of the MFI of cytosolic dsDNA per cell in the indicated UM-SCC-1 stable cells; *n* = 68 (pBabe-pLVX), 176 (R273H-pLVX), 182 (R273H-MCM5). **e**, Dot plot of the relative cytosolic dsDNA concentration of the indicated UM-SCC-1 stable cells. *n* = 3 in each group. **f**, Representative IF staining images of cytosolic ssDNA in UM-SCC-1 stable cells with or without S1 nuclease. Note that ssDNA staining was eliminated by S1 nuclease treatment. **g**, Representative IF staining images of cytosolic ssDNA in the indicated UM-SCC-1 stable cell lines treated with or without HU (150 μM, 48 h). **h**, Violin plot of the MFI of cytosolic ssDNA per cell in the indicated UM-SCC-1 stable cells in the absence or presence of HU; *n* = 203 (pBabe-pLVX), 346 (pBabe-pLVX + HU), 303 (pBabe-MCM5), 328 (pBabe-MCM5 + HU), 216 (G245D-pLVX), 400 (G245D-pLVX + HU), 198 (G245D-MCM5), 327 (G245D-MCM5 + HU). **i** and **j**, Dot plots of the relative cytosolic dsDNA concentration of the indicated stable cells. *n* = 3 in each group. **k** and **m**, Representative IF staining images of cytosolic ssDNA in the indicated stable cell lines in the absence or presence of HU (100 μM, 48 h). Scale bar, 10 μm (**b**, **c**, **f**, **g**, **k** and **m**). **l** and **n**, Violin plots of the MFI of cytosolic ssDNA per cell in the indicated stable cells; *n* = 110 (Lenti-ctrl-shNT), 164 (Lenti-ctrl-shNT + HU), 136 (shp53-shNT), 111 (shp53-shNT + HU), 108 (shp53-shMCM5-C), 128 (shp53-shMCM5-C + HU), 107 (shp53-shMCM5-G), 266 (shp53-shMCM5-G + HU) (**l**); *n* = 343 (Lenti-ctrl-shNT), 254 (Lenti-ctrl-shNT + HU), 343 (shp53-shNT), 357 (shp53-shNT + HU), 189 (shp53-shMCM5-C), 208 (shp53-shMCM5-C + HU), 204 (shp53-shMCM5-G), 202 (shp53-shMCM5-G + HU) (**n**). **o,** Intracellular cGAMP concentration of the indicated UM-SCC-1 stable cell lines in the absence or presence of HU. *n* = 3 in each group. **p-s**, Western blot analyses of the cytosolic and/or nuclear fractions from the indicated stable cells. Cells were incubated with doxycycline (200 ng/mL) for 48 h before further HU treatment (**k**)-(**n**), and (**s**). Bars represent the median ± quartiles (**d**), (**h**), (**l**), and (**n**), mean ± SD (**e**), (**i**), (**j**), and (**o**). Significances were tested by the Kruskal-Wallis test with Dunn's multiple comparisons test (**d**), (**h**), and (**n**) or by one-way ANOVA with Tukey's multiple comparisons test (**e**), (**i**), (**j**), (**l**) and (**o**). Source data are provided as a Source Data file.

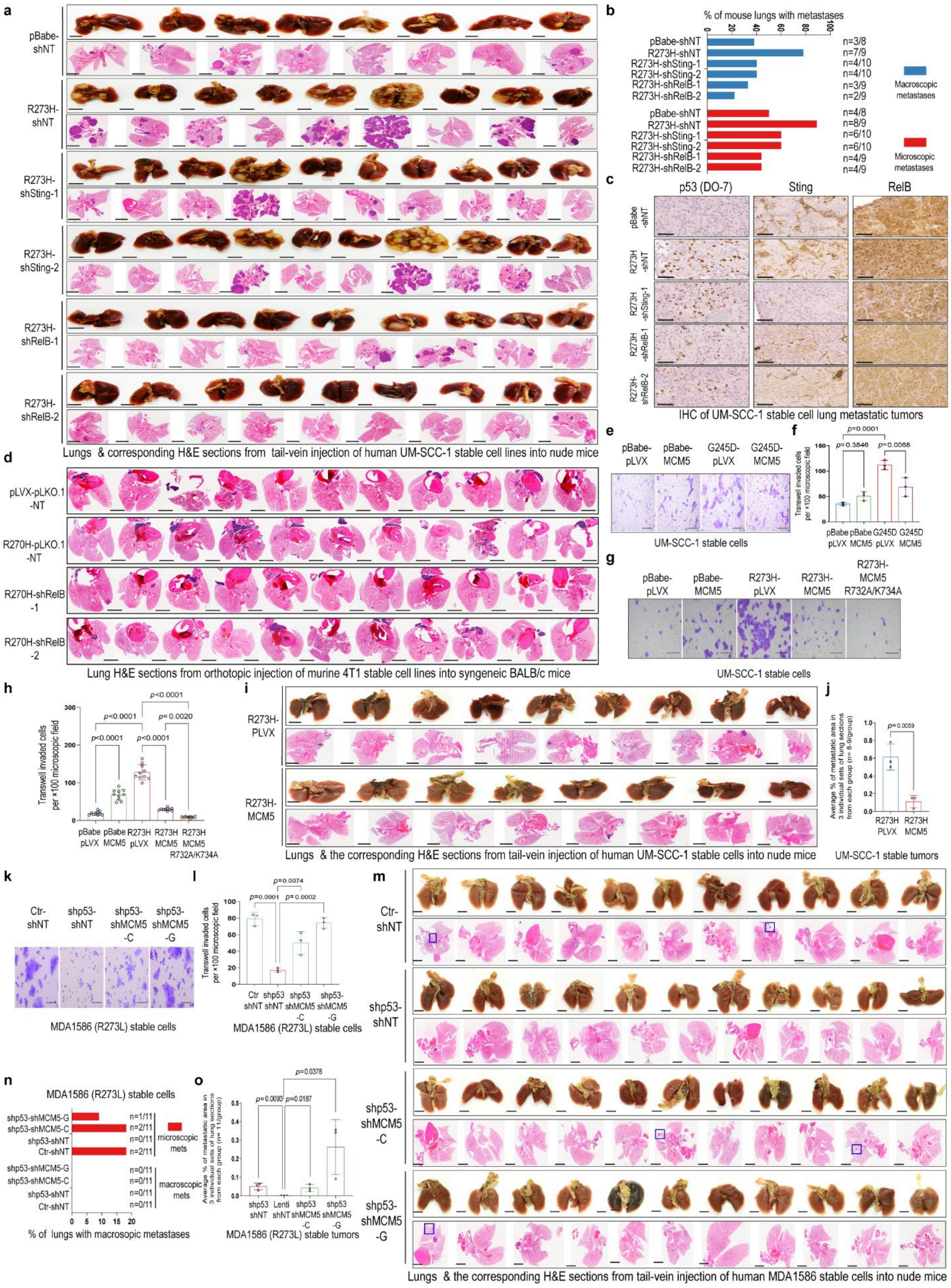
a
pBabe-shNT
R273H-shNT
R273H-shSting-1
R273H-shSting-2
R273H-shRelB-1
R273H-shRelB-2
Lungs & corresponding H&E sections from tail-vein injection of human UM-SCC-1 stable cell lines into nude mice
b
% of mouse lungs with metastases
pBabe-shNT n=3/8
R273H-shNT n=7/9
R273H-shSting-1 n=4/10
R273H-shSting-2 n=4/10
R273H-shRelB-1 n=3/9
R273H-shRelB-2 n=2/9
Macroscopic metastases
pBabe-shNT n=4/8
R273H-shNT n=8/9
R273H-shSting-1 n=6/10
R273H-shSting-2 n=6/10
R273H-shRelB-1 n=4/9
R273H-shRelB-2 n=4/9
Microscopic metastases
c
p53 (DO-7)
Sting
RelB
IHC of UM-SCC-1 stable cell lung metastatic tumors
d
pLVX-pLKO.1-NT
R270H-pLKO.1-NT
R270H-shRelB-1
R270H-shRelB-2
Lung H&E sections from orthotopic injection of murine 4T1 stable cell lines into syngeneic BALB/c mice
e
pBabe-pLVX
pBabe-MCM5
G245D-pLVX
G245D-MCM5
UM-SCC-1 stable cells
f
p=0.0001
p=0.3846
p=0.0068
Transwell invaded cells per ×100 microscopic field
pBabe pLVX
pBabe MCM5
G245D pLVX
G245D MCM5
g
pBabe-pLVX
pBabe-MCM5
R273H-pLVX
R273H-MCM5
R273H-MCM5 R732A/K734A
UM-SCC-1 stable cells
h
p<0.0001
p<0.0001
p=0.0020
p<0.0001
p<0.0001
Transwell invaded cells per ×100 microscopic field
pBabe pLVX
pBabe MCM5
R273H pLVX
R273H MCM5
R273H MCM5 R732A/K734A
i
R273H-PLVX
R273H-MCM5
Lungs & the corresponding H&E sections from tail-vein injection of human UM-SCC-1 stable cells into nude mice
j
p=0.0059
Average % of metastatic area in 3 individual sets of lung sections from each group (n= 8-9/group)
R273H PLVX
R273H MCM5
UM-SCC-1 stable tumors
k
Ctr-shNT
shp53-shNT
shp53-shMCM5-C
shp53-shMCM5-G
MDA1586 (R273L) stable cells
l
p=0.0074
p=0.0001
p=0.0002
Transwell invaded cells per ×100 microscopic field
Ctr shNT
shp53 shNT
shp53 shMCM5 -C
shp53-shMCM5 -G
MDA1586 (R273L) stable cells
m
Ctr-shNT
shp53-shNT
shp53-shMCM5 -C
shp53-shMCM5 -G
Lungs & the corresponding H&E sections from tail-vein injection of human MDA1586 stable cells into nude mice
n
MDA1586 (R273L) stable cells
shp53-shMCM5-G n=1/11
shp53-shMCM5-C n=2/11
shp53-shNT n=0/11
Ctr-shNT n=2/11
microscopic mets
shp53-shMCM5-G n=0/11
shp53-shMCM5-C n=0/11
shp53-shNT n=0/11
Ctr-shNT n=0/11
macroscopic mets
% of lungs with macrosopic metastases
o
p=0.0378
p=0.0093
p=0.0187
Average % of metastatic area in 3 individual sets of lung sections from each group (n= 11/group)
shp53 shNT
Lenti shNT
shp53 shMCM5 -C
shp53 shMCM5 -G
MDA1586 (R273L) stable tumors

Supplementary Fig. 4: GOF mutp53-MCM5-STING-NC-NF-κB signaling promotes tumor cell migration, invasion and metastasis

a, Macroscopic images of lungs and microscopic images of the corresponding H & E-stained sections from nude mice 18 weeks after tail-vein injection with the indicated UM-SCC-1 stable cell lines (10^6 cells/mouse). Scale bar, 6 mm. **b**, Summary graph showing percentage of mouse lungs with macroscopic and microscopic metastases in the experiment in (**a**). **c**, Representative images of immunohistochemistry (IHC) staining of lung metastatic lesions from (**a**) as indicated. Scale bar, 60 μm. Note that the R273H mutp53-expressing tumor (R273H-shNT) had a higher ratio of nuclear RelB staining than did the pBabe control, but this was decreased by *STING* or *RelB* knockdown. **d**, Microscopic images of the H & E-stained lung sections from BALB/c mice 4 weeks after mammary fat pad injection with the indicated mouse 4T1 stable cell lines (1.5×10^5 cells/mouse). Scale bar, 6 mm. **e** and **g**, Representative images of Transwell migration and invasion assays of cell lines as indicated. Scale bar, 100 μm. **f** and **h**, Summary graphs of the indicated invading stable cell lines; $n = 3$ in each group (**f**), $n = 10$ in each group (**h**). error bars represent SD. Significances were calculated by one-way ANOVA with Tukey's multiple comparisons test. pBabe and pLVX, control vectors. **i**, Representative macroscopic images of lungs and microscopic images of the corresponding microscopic H & E-stained sections from mice 12 weeks after tail-vein injection with UM-SCC-1 stable cell lines (5×10^5 cells/mouse). Scale bar, 6 mm. **j**, Mean percentage of lung metastatic areas from (**i**). $n = 3$ in each group. Shown are mean percentages per group of lung metastatic areas from 3 consecutive sections in each lung, separated by 200 μm. See the Methods for details. Error bars represent SD. Significance tested using two-tailed unpaired *t*-test. **k** and **l**, *MCM5* knockdown rescues impaired invasion caused by R273L mutp53 knockdown in MDA1586 stable cells. Shown are representative images (**k**) and a summary graph of Transwell invasion assays (**l**); Scale bar, 100 μm (**k**), $n = 3$ in each group (**l**). error bars represent SD; Significances were calculated by one-way ANOVA with Tukey's multiple comparisons test (**l**). **m**, *MCM5* knockdown rescues impaired lung metastasis induced by R273L mutp53 downregulation in MDA1586 stable cells. Shown are macroscopic images of lungs and the corresponding microscopic H & E-stained sections from nude mice 6 months after tail-vein injection with MDA1586 stable cell lines (0.77×10^6 cells/mouse). Scale bar, 6 mm. Positive MDA1586 microscopic metastatic lesions are marked by the blue squares. **n**, Percentage of lung area with macroscopic and microscopic metastases from (**m**). **o**, Mean percentage of lung tumor metastatic areas from (**m**). $n = 3$ in each group. Shown are the group mean percentages of lung area with metastatic tumors from 3 random sections per lung, each 100 μm apart. See the Methods for details. Error bars represent SD; Significances were calculated by the two-tailed unpaired *t*-test. Source data are provided as a Source Data file.

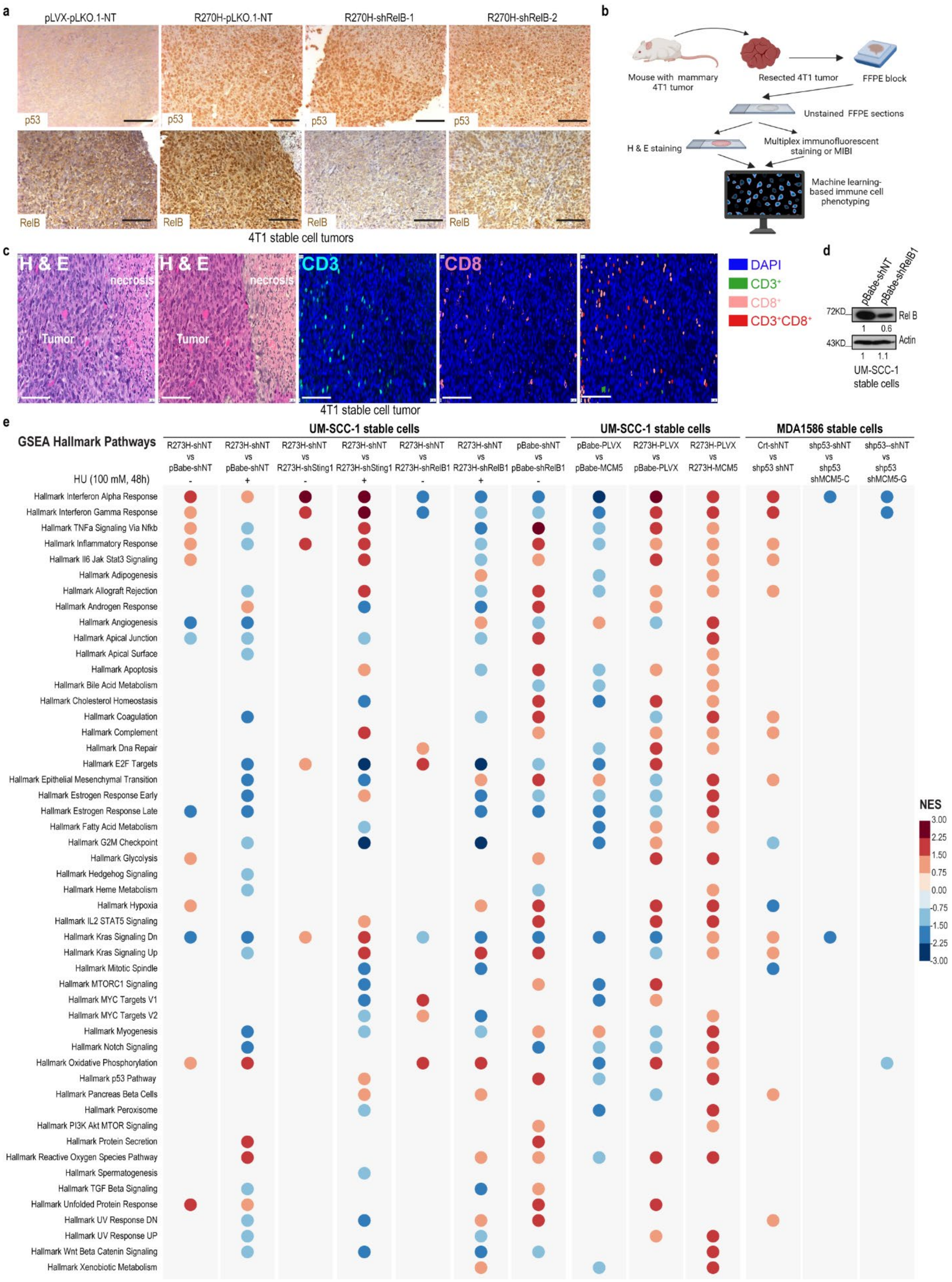
a
pLVX-pLKO.1-NT
R270H-pLKO.1-NT
R270H-shRelB-1
R270H-shRelB-2
p53
RelB
4T1 stable cell tumors
b
Mouse with mammary 4T1 tumor
Resected 4T1 tumor
FFPE block
Unstained FFPE sections
H & E staining
Multiplex immunofluorescent staining or MIBI
Machine learning-based immune cell phenotyping
c
H & E
necrosis
Tumor
CD3
CD8
DAPI
CD3+
CD8+
CD3+CD8+
4T1 stable cell tumor
d
pBabe-shNT
pBabe-shRelB1
72KD
Rel B
1 0.6
43KD
Actin
1 1.1
UM-SCC-1 stable cells
e
GSEA Hallmark Pathways
UM-SCC-1 stable cells
UM-SCC-1 stable cells
MDA1586 stable cells
R273H-shNT vs pBabe-shNT
R273H-shNT vs pBabe-shNT
R273H-shNT vs R273H-shSting1
R273H-shNT vs R273H-shSting1
R273H-shNT vs R273H-shRelB1
R273H-shNT vs R273H-shRelB1
pBabe-shNT vs pBabe-shRelB1
pBabe-PLVX vs pBabe-MCM5
R273H-PLVX vs pBabe-PLVX
R273H-PLVX vs R273H-MCM5
Crt-shNT vs shp53 shNT
shp53-shNT vs shp53 shMCM5-C
shp53--shNT vs shp53 shMCM5-G
HU (100 mM, 48h)
- + - + - + -
Hallmark Interferon Alpha Response
Hallmark Interferon Gamma Response
Hallmark TNFa Signaling Via Nfkb
Hallmark Inflammatory Response
Hallmark Il6 Jak Stat3 Signaling
Hallmark Adipogenesis
Hallmark Allograft Rejection
Hallmark Androgen Response
Hallmark Angiogenesis
Hallmark Apical Junction
Hallmark Apical Surface
Hallmark Apoptosis
Hallmark Bile Acid Metabolism
Hallmark Cholesterol Homeostasis
Hallmark Coagulation
Hallmark Complement
Hallmark Dna Repair
Hallmark E2F Targets
Hallmark Epithelial Mesenchymal Transition
Hallmark Estrogen Response Early
Hallmark Estrogen Response Late
Hallmark Fatty Acid Metabolism
Hallmark G2M Checkpoint
Hallmark Glycolysis
Hallmark Hedgehog Signaling
Hallmark Heme Metabolism
Hallmark Hypoxia
Hallmark IL2 STAT5 Signaling
Hallmark Kras Signaling Dn
Hallmark Kras Signaling Up
Hallmark Mitotic Spindle
Hallmark MTORC1 Signaling
Hallmark MYC Targets V1
Hallmark MYC Targets V2
Hallmark Myogenesis
Hallmark Notch Signaling
Hallmark Oxidative Phosphorylation
Hallmark p53 Pathway
Hallmark Pancreas Beta Cells
Hallmark Peroxisome
Hallmark PI3K Akt MTOR Signaling
Hallmark Protein Secretion
Hallmark Reactive Oxygen Species Pathway
Hallmark Spermatogenesis
Hallmark TGF Beta Signaling
Hallmark Unfolded Protein Response
Hallmark UV Response DN
Hallmark UV Response UP
Hallmark Wnt Beta Catenin Signaling
Hallmark Xenobiotic Metabolism
NES
3.00
2.25
1.50
0.75
0.00
-0.75
-1.50
-2.25
-3.00

Supplementary Fig. 5: RelB IHC, T cell multiplex immunofluorescent staining, and GSEA
a, Representative IHC staining images of p53 and RelB from 4T1 stable cell tumors from BALB/c mice as indicated. Scale bar, 89.7 μm. **b**, Schematic representation of multiplexed IHC and MIBI analyses. Created with BioRender.com. FFPE, formalin-fixed paraffin-embedded. **c**, Representative H & E staining images and their corresponding multiplex CD3 and CD8 immunofluorescent and phenotyping images from a 4T1 stable cell tumor from BALB/c mouse. Scale bar, 100 μm. **d**, Western blot analysis of UM-SCC-1 stable cell lines as indicated**. e**, Summary of normalized enrichment scores (NES) for all the GSEA Hallmark pathways significantly enriched (FDRq < 25%) in the indicated comparisons of cell lines. +HU: 100 μM, 48 h. Source data are provided as a Source Data file.

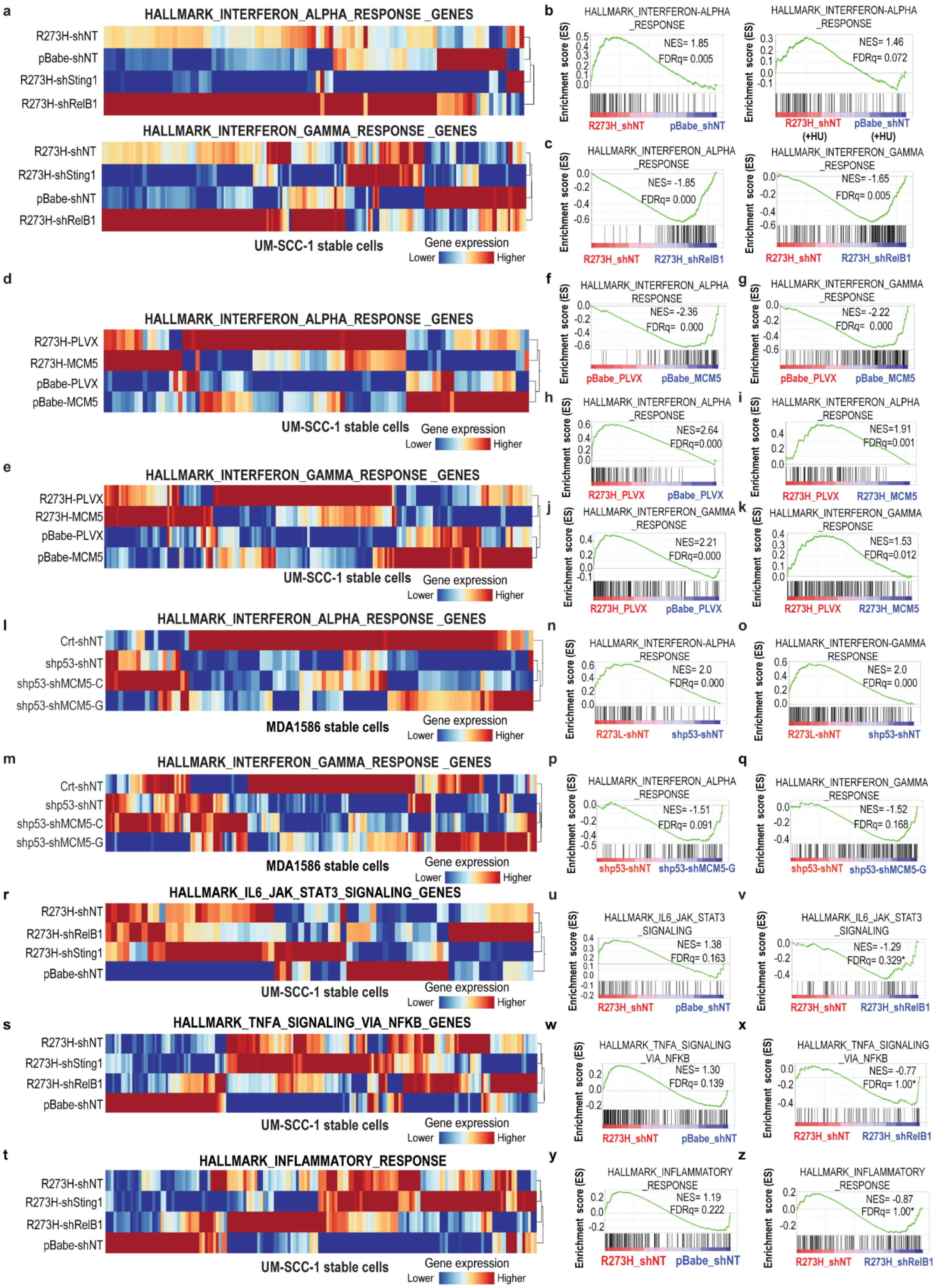
a
HALLMARK_INTERFERON_ALPHA_RESPONSE _GENES
R273H-shNT
pBabe-shNT
R273H-shSting1
R273H-shRelB1
HALLMARK_INTERFERON_GAMMA_RESPONSE _GENES
R273H-shNT
R273H-shSting1
pBabe-shNT
R273H-shRelB1
UM-SCC-1 stable cells
Gene expression
Lower
Higher
b
HALLMARK_INTERFERON-ALPHA _RESPONSE
Enrichment score (ES)
NES= 1.85
FDRq= 0.005
R273H_shNT
pBabe_shNT
HALLMARK_INTERFERON-ALPHA _RESPONSE
NES= 1.46
FDRq= 0.072
R273H_shNT (+HU)
pBabe_shNT (+HU)
c
HALLMARK_INTERFERON_ALPHA _RESPONSE
NES= -1.85
FDRq= 0.000
R273H_shNT
R273H_shRelB1
HALLMARK_INTERFERON_GAMMA _RESPONSE
NES= -1.65
FDRq= 0.005
R273H_shNT
R273H_shRelB1
d
HALLMARK_INTERFERON_ALPHA_RESPONSE _GENES
R273H-PLVX
R273H-MCM5
pBabe-PLVX
pBabe-MCM5
UM-SCC-1 stable cells
Gene expression
Lower
Higher
e
HALLMARK_INTERFERON_GAMMA_RESPONSE _GENES
R273H-PLVX
R273H-MCM5
pBabe-PLVX
pBabe-MCM5
UM-SCC-1 stable cells
Gene expression
Lower
Higher
f
HALLMARK_INTERFERON_ALPHA RESPONSE
NES= -2.36
FDRq= 0.000
pBabe_PLVX
pBabe_MCM5
g
HALLMARK_INTERFERON_GAMMA _RESPONSE
NES= -2.22
FDRq= 0.000
pBabe_PLVX
pBabe_MCM5
h
HALLMARK_INTERFERON_ALPHA _RESPONSE
NES=2.64
FDRq=0.000
R273H_PLVX
pBabe_PLVX
i
HALLMARK_INTERFERON_ALPHA _RESPONSE
NES=1.91
FDRq=0.001
R273H_PLVX
R273H_MCM5
j
HALLMARK_INTERFERON_GAMMA _RESPONSE
NES=2.21
FDRq=0.000
R273H_PLVX
pBabe_PLVX
k
HALLMARK_INTERFERON_GAMMA _RESPONSE
NES=1.53
FDRq=0.012
R273H_PLVX
R273H_MCM5
l
HALLMARK_INTERFERON_ALPHA_RESPONSE _GENES
Crt-shNT
shp53-shNT
shp53-shMCM5-C
shp53-shMCM5-G
MDA1586 stable cells
Gene expression
Lower
Higher
m
HALLMARK_INTERFERON_GAMMA_RESPONSE _GENES
Crt-shNT
shp53-shNT
shp53-shMCM5-C
shp53-shMCM5-G
MDA1586 stable cells
Gene expression
Lower
Higher
n
HALLMARK_INTERFERON-ALPHA _RESPONSE
NES= 2.0
FDRq= 0.000
R273L-shNT
shp53-shNT
o
HALLMARK_INTERFERON-GAMMA RESPONSE
NES= 2.0
FDRq= 0.000
R273L-shNT
shp53-shNT
p
HALLMARK_INTERFERON_ALPHA _RESPONSE
NES= -1.51
FDRq= 0.091
shp53-shNT
shp53-shMCM5-G
q
HALLMARK_INTERFERON_GAMMA _RESPONSE
NES= -1.52
FDRq= 0.168
shp53-shNT
shp53-shMCM5-G
r
HALLMARK_IL6_JAK_STAT3_SIGNALING_GENES
R273H-shNT
R273H-shRelB1
R273H-shSting1
pBabe-shNT
UM-SCC-1 stable cells
Gene expression
Lower
Higher
s
HALLMARK_TNFA_SIGNALING_VIA_NFKB_GENES
R273H-shNT
R273H-shSting1
R273H-shRelB1
pBabe-shNT
UM-SCC-1 stable cells
Gene expression
Lower
Higher
t
HALLMARK_INFLAMMATORY_RESPONSE
R273H-shNT
R273H-shSting1
R273H-shRelB1
pBabe-shNT
UM-SCC-1 stable cells
Gene expression
Lower
Higher
u
HALLMARK_IL6_JAK_STAT3 _SIGNALING
NES= 1.38
FDRq= 0.163
R273H_shNT
pBabe_shNT
v
HALLMARK_IL6_JAK_STAT3 _SIGNALING
NES= -1.29
FDRq= 0.329*
R273H_shNT
R273H_shRelB1
w
HALLMARK_TNFA_SIGNALING _VIA_NFKB
NES= 1.30
FDRq= 0.139
R273H_shNT
pBabe_shNT
x
HALLMARK_TNFA_SIGNALING _VIA_NFKB
NES= -0.77
FDRq= 1.00*
R273H_shNT
R273H_shRelB1
y
HALLMARK_INFLAMMATORY _RESPONSE
NES= 1.19
FDRq= 0.222
R273H_shNT
pBabe_shNT
z
HALLMARK_INFLAMMATORY _RESPONSE
NES= -0.87
FDRq= 1.00*
R273H_shNT
R273H_shRelB1

Supplementary Fig. 6: GOF mutp53-MCM5-STING-NC-NF-κB signaling antagonizes IFN signaling and regulates inflammation-related gene expression

a, Hierarchical clustering analyses of the Hallmark IFNα response gene set (97 genes, x-axis) and the Hallmark IFNγ response gene set (200 genes) from the comparisons of UM-SCC-1 stable cell lines as indicated. **b** and **c**, GSEA of Hallmark IFNα and IFNγ response signaling pathway gene sets from comparisons of the indicated UM-SCC-1 stable cell lines treated without or with hydroxyurea (+HU: 100 μM, 48 h). **d** and **e**, Hierarchical clustering analyses of the Hallmark IFNα response gene set (97 genes, x-axis) (**d**) and IFNγ response gene set (200 genes) (**e**) from the indicated comparisons of UM-SCC-1 stable cell lines. pLVX, empty vector. **f**-**k**, GSEA of Hallmark IFNα and IFNγ response signaling pathway gene sets from the indicated comparisons of UM-SCC-1 stable cell lines. **l** and **m**, Hierarchical clustering analyses of the Hallmark IFNα response gene set (97 genes, x-axis) (**l**) and IFNγ response gene set (200 genes) (**m**) from the indicated comparisons of MDA1586 stable cell lines. NT, non-target. **n**-**q**, GSEA of Hallmark IFNα and IFNγ response signaling gene sets from the indicated comparisons of MDA1586 stable cell lines. **r**-**t**, Hierarchical clustering analyses of the Hallmark IL6-JAK-STAT3 signaling gene set (87 genes, x-axis) (**r**), the Hallmark TNFα signaling via NF-κB gene set (200 genes, x-axis) (**s**), and the Hallmark Inflammatory response gene set (200 genes, x-axis) (**t**) from the indicated comparisons of UM-SCC-1 stable cell lines. **u**-**z**, GSEA of Hallmark IL6-JAK-STAT3, TNFα signaling via NF-κB, and Inflammatory response gene sets from the comparisons of the indicated UM-SCC-1 stable cell lines. NT, non-target. *FDRq > 25%, not significant. Source data are provided as a Source Data file.

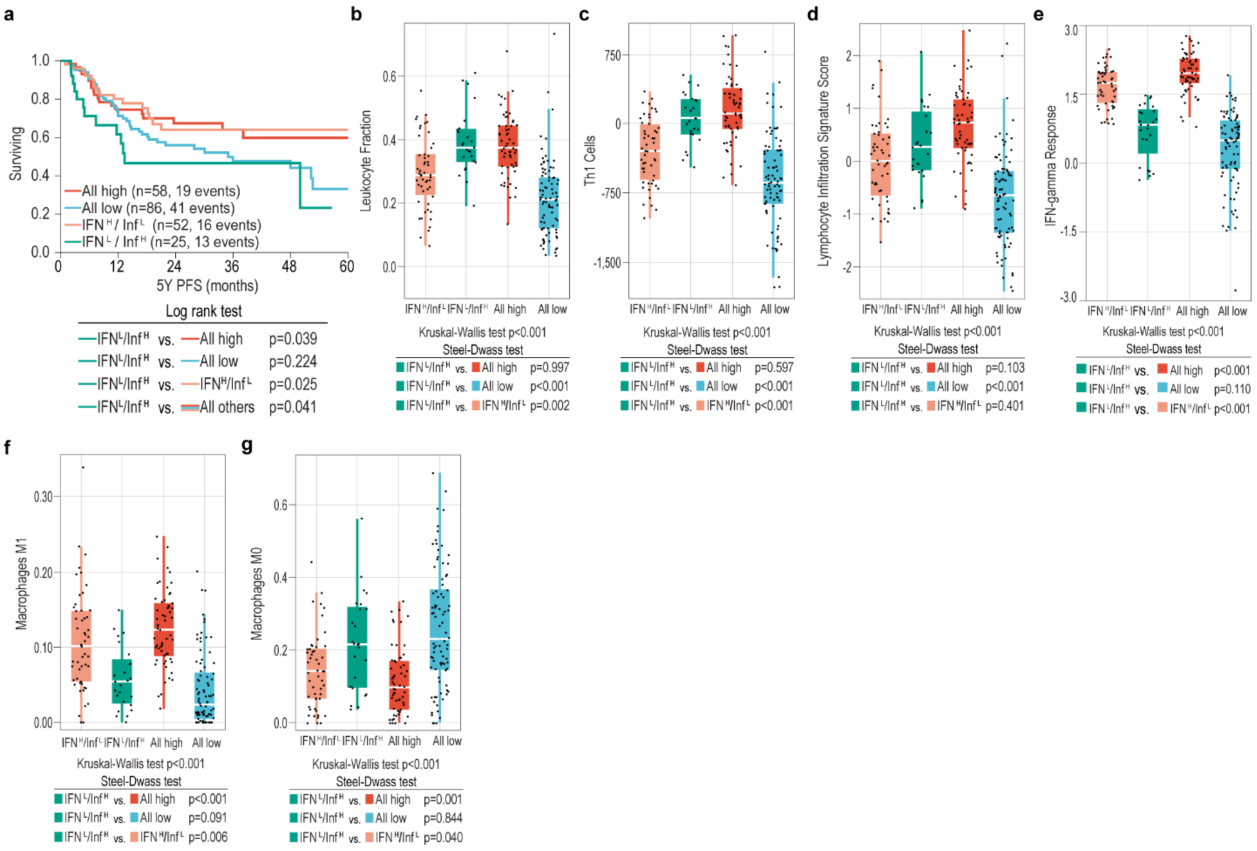

Supplementary Fig. 7: The IFN^{L}/Inf^{H} gene signature is associated with worse clinical outcomes and immunosuppression in OSCCs

a, Five-year progression-free survival (PFS) for the 221 patients with HPV-negative *TP53*-mutant OSCC with the indicated IFN and Inf gene expression profiles. L, low; H, high. **b-g**, Box plots of immune enrichment scores of 221 *TP53*-mutant OSCCs with the indicated IFN and inflammation-related (Inf) gene expression profiles. The number of tumors in each group were: IFN^{H}/Inf^{L}=52, IFN^{L}/Inf^{H} = 25, All High = 58, and All Low = 86. The mean immune enrichment scores (± SD) were: (**b**) IFN^{H}/Inf^{L}: 0.29 (± 0.11), IFN^{L}/Inf^{H}: 0.39 (± 0.1), All High: 0.38 (± 0.1), All Low: 0.22 (± 0.12); (**c**) IFN^{H}/Inf^{L}: -297.72 (± 350.82), IFN^{L}/Inf^{H}: 54.47 (± 270.32), All High: 155 (± 372.54), All low: -609.67 (± 497.7); (**d**) IFN^{H}/Inf^{L}: -0.01 (± 0.79) , IFN^{L}/Inf^{H}: 0.3 (± 0.71), All High: 0.66 (± 0.69), All Low: -0.68 (± 0.89). (**e**) IFN^{H}/Inf^{L}: 1.67 (±0.44), IFN^{L}/Inf^{H}: 0.71 (±0.6), All High: 1.97 (±0.44), All Low: 0.3 (±0.85). (**f**) IFN^{H}/Inf^{L}: 0.11 (± 0.07), IFN^{L}/Inf^{H}: 0.06 (± 0.04), All High: 0.12 (± 0.05), All Low: 0.04 (± 0.05); (**g**) IFN^{H}/Inf^{L}: 0.14 (± 0.1), IFN^{L}/Inf^{H}: 0.22 (± 0.13), All High: 0.11 (± 0.09), All Low: 0.26 (±0.17). Boxes represent the IQR and the horizontal line indicates the median. The whiskers extend to the last data point within 1.5×IQR. Significances were tested using Kruskal-Wallis test and two-sided steel-dwass test (**b-g**). Source data are provided as a Source Data file.

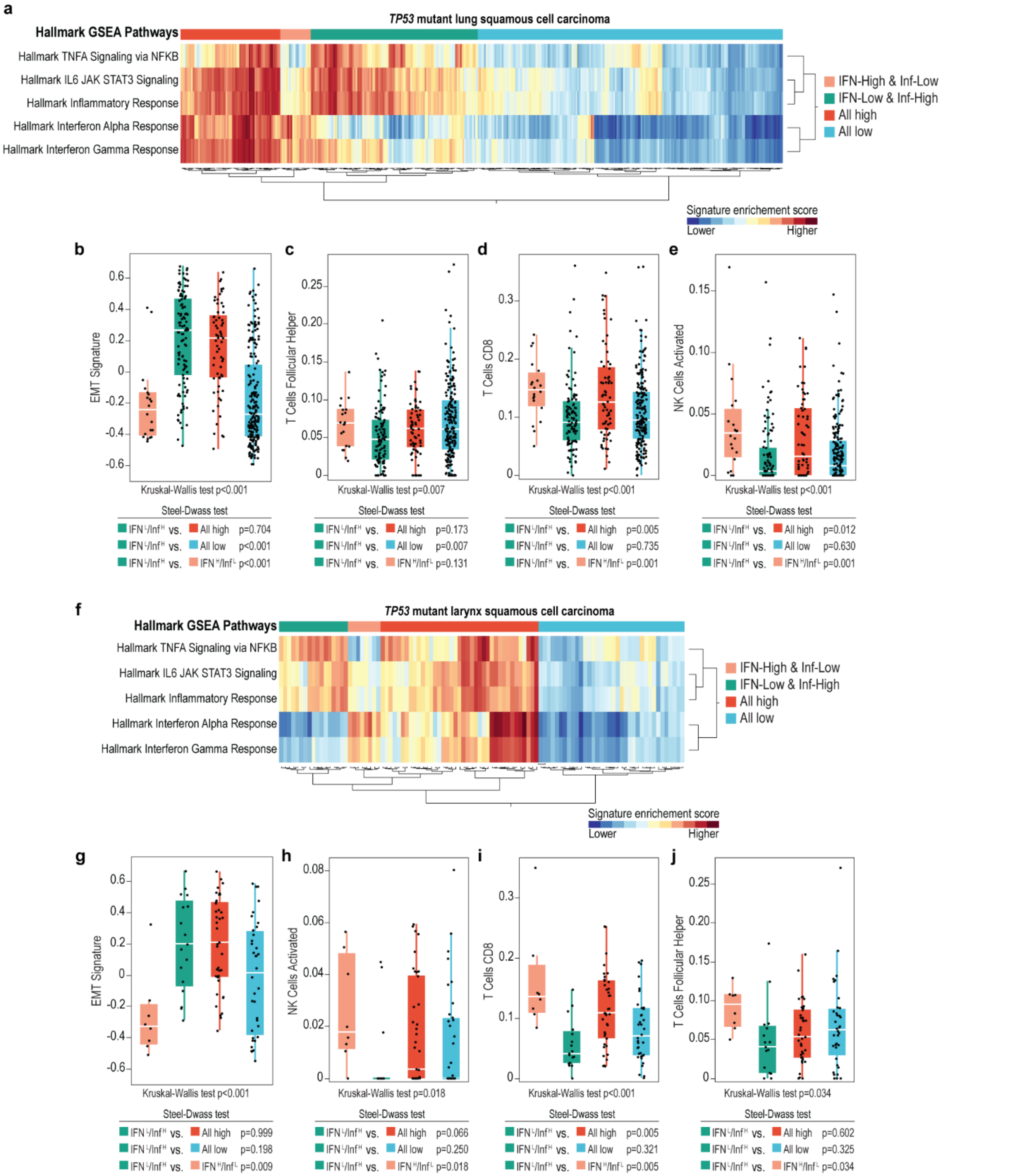

Supplementary Fig. 8: The IFN^{L}/Inf^{H} gene signature is associated with immunosuppression in lung and larynx squamous cell carcinomas

a, Hierarchical clustering analysis of single-sample GSEA scores for the Hallmark GSEA IFN and inflammation-related (Inf) signaling pathways for 399 TCGA patients with HPV-negative, *TP53*-mutant lung squamous cell carcinoma (x-axis). The analysis revealed 4 patient groups with distinct gene expression profiles. **b-e**, Box plots of EMT and immune enrichment scores of the 399 HPV-

negative and *TP53*-mutant lung squamous cell carcinoma with the indicated IFN and Inf gene expression profiles. The number of patients in each group were IFN^H/Inf^L = 20, IFN^L/Inf^H = 111, All High = 66, and All Low = 202. The mean immune enrichment scores (± SD) were: (**b**) IFN^H/Inf^L: -0.25 (±0.24), IFN^L/Inf^H: 0.20 (±0.30), All High: 0.16 (±0.28), All Low: -0.18 (±0.30); (**c**) IFN^H/Inf^L: 0.07 (±0.03), IFN^L/Inf^H: 0.05 (±0.04), All High: 0.06 (±0.03), All Low: 0.07 (±0.05); (**d**) IFN^H/Inf^L: 0.15 (±0.05), IFN^L/Inf^H: 0.10 (±0.06), All High: 0.14 (±0.08), All Low: 0.11 (±0.06); (**e**) IFN^H/Inf^L: 0.04 (±0.04), IFN^L/Inf^H: 0.02 (±0.03), All High: 0.03 (±0.03), All Low: 0.02 (±0.03); Boxes represent the IQR and the horizontal line indicates the median. The whiskers extend to the last data point within 1.5×IQR. Significances were tested using Kruskal-Wallis test and two-sided steel-dwass test (**b**-**e**) **f**, Hierarchical clustering analysis of single-sample GSEA scores for the Hallmark GSEA IFN and inflammation-related (Inf) signaling pathways for 100 TCGA patients with HPV-negative, *TP53*-mutant larynx squamous cell carcinoma (x-axis). The analysis revealed 4 patient groups with distinct gene expression profiles. **g-j**, Box plots of EMT and immune enrichment scores of the 100 HPV-negative and *TP53*-mutant larynx squamous cell carcinoma with the indicated IFN and Inf gene expression profiles. The number of patients in each group were IFN^H/Inf^L = 8, IFN^L/Inf^H = 17, All High = 39, and All Low = 36. The mean immune enrichment scores (± SD) were: (**g**) IFN^H/Inf^L: -0.27 (±0.26), IFN^L/Inf^H: 0.20 (±0.30), All High: 0.20 (±0.29), All Low: -0.02 (±0.35); (**h**) IFN^H/Inf^L: 0.09 (±0.03), IFN^L/Inf^H: 0.05 (±0.05), All High: 0.06 (±0.04), All Low: 0.07 (±0.05); (**i**) 0.16 (±0.08), IFN^L/Inf^H: 0.06 (±0.04), All High: 0.12 (±0.06), All Low: 0.08 (±0.05); (**j**) IFN^H/Inf^L: 0.03 (±0.02), IFN^L/Inf^H: 0.01 (±0.01), All High: 0.02 (±0.02), All Low: 0.01 (±0.02); Boxes represent the IQR and the horizontal line indicates the median. The whiskers extend to the last data point within 1.5×IQR. Significances were tested using Kruskal-Wallis test and two-sided steel-dwass test (**g**-**j**). Source data are provided as a Source Data file.
