# Supplementary material for: Mutant p53 gains oncogenic functions through a chromosomal instability-induced cytosolic DNA response
Source: Nat Commun. 2024 Jan 2;15:180. doi: 10.1038/s41467-023-44239-2 (PMC10761733; doi:10.1038/s41467-023-44239-2)
Supplement: Supplementary file 3 — Description of Additional Supplementary Files [file 41467_2023_44239_MOESM3_ESM.pdf]

Description of Additional Supplementary Files

File Name: Supplementary Data 1
Description: G245D mutp53 interactome identified by SILAC-IP MS from 8 individual experiments

File Name: Supplementary Data 2
Description: Cell line genotype STR information

File Name: Supplementary Data 3
Description: DNA vector, primer and shRNA information

File Name: Supplementary Data 4
Description: Antibody information
