# Supplementary material for: Mutant p53 gains oncogenic functions through a chromosomal instability-induced cytosolic DNA response
Source: Nat Commun. 2024 Jan 2;15:180. doi: 10.1038/s41467-023-44239-2 (PMC10761733; doi:10.1038/s41467-023-44239-2)
Supplement: Supplementary file 8 — Reporting Summary [file 41467_2023_44239_MOESM8_ESM.pdf]

nature portfolio

Corresponding author(s): Jeffrey N Myers & Ge Zhou

Last updated by author(s): 11/29/2023

Reporting Summary

Nature Portfolio wishes to improve the reproducibility of the work that we publish. This form provides structure for consistency and transparency in reporting. For further information on Nature Portfolio policies, see our Editorial Policies and the Editorial Policy Checklist.

Statistics

For all statistical analyses, confirm that the following items are present in the figure legend, table legend, main text, or Methods section.

n/a	Confirmed	
☐	☒	The exact sample size (*n*) for each experimental group/condition, given as a discrete number and unit of measurement
☐	☒	A statement on whether measurements were taken from distinct samples or whether the same sample was measured repeatedly
☐	☒	The statistical test(s) used AND whether they are one- or two-sided<br>*Only common tests should be described solely by name; describe more complex techniques in the Methods section.*
☐	☒	A description of all covariates tested
☐	☒	A description of any assumptions or corrections, such as tests of normality and adjustment for multiple comparisons
☐	☒	A full description of the statistical parameters including central tendency (e.g. means) or other basic estimates (e.g. regression coefficient) AND variation (e.g. standard deviation) or associated estimates of uncertainty (e.g. confidence intervals)
☐	☒	For null hypothesis testing, the test statistic (e.g. *F*, *t*, *r*) with confidence intervals, effect sizes, degrees of freedom and *P* value noted<br>*Give P values as exact values whenever suitable.*
☒	☐	For Bayesian analysis, information on the choice of priors and Markov chain Monte Carlo settings
☐	☒	For hierarchical and complex designs, identification of the appropriate level for tests and full reporting of outcomes
☒	☐	Estimates of effect sizes (e.g. Cohen's *d*, Pearson's *r*), indicating how they were calculated

Our web collection on statistics for biologists contains articles on many of the points above.

Software and code

Policy information about availability of computer code

Data collection	Nikon Eclipse TE100, Andor Revolution XDi WD spinning disk confocal microscope and Vectra Polaris imaging system (Akoya Biosciences) were used for image captures. Multiplexed ion beam imaging (MIBI) technology and service provided by IONPATH (Menlo Park, California). Illumina HiSeq4000 sequencer provided by Novogene was used for RNAseq. TCGA whole exome sequencing (MC3 MAF files) and clinical data were retrieved using the softwares RStudio (RStudio 2022.07.2+576 "Spotted Wakerobin" for macOS) and the R packages MAFtools (version 2.12.0) (https://github.com/PoisonAlien/maftools) and PoisonAlien/TCGAmutations (https://github.com/PoisonAlien/TCGAmutations). RNAseq data were recovered from the National Cancer Institute's Genomic Data Commons (GDC) (https://portal.gdc.cancer.gov/). Genomic and clinicopathological data from squamous carcinomas were from TCGA (https://gdc.cancer.gov/about-data/publications/PanCan-Squamous-2018).
Data analysis	ImageJ (V1.8.0), Imaris × 64 9.3.0, Visiopharm (2022.03), GraphPad Prism 9 and JMP Pro 15.0 were used for image and statistical analyses. The quality of the raw RNAseq FASTQ files was checked with the FASTQC package (https://www.bioinformatics.babraham.ac.uk/projects/fastqc/). The TopHat package (2.1.1) or STAR (2.7.9a), Samtools software (1.8), HTSeq software (0.11.0) and DESeq (1.34.0) package for R were used to align, sort, count and normalize RNAseq data. TCGA Genomic data analyses was performed using the JMP Pro 15.0 Software and with the software RStudio (RStudio 2022.07.2+576 "Spotted Wakerobin" for macOS) and the R packages MAFtools (version 2.12.0) (https://github.com/PoisonAlien/maftools). Gene set enrichment analyses (GSEA) and single sample gene set enrichment analyses (ssGSEA) were performed together with analysis of the molecular signatures database (MSigDB) (https://www.gsea-msigdb.org/gsea/index.jsp) using the Hallmark gene set (h.all.v7.0.symbols.gmt) (https://www.gsea-msigdb.org/gsea/msigdb/human/genesets.jsp?collection=H).

For manuscripts utilizing custom algorithms or software that are central to the research but not yet described in published literature, software must be made available to editors and reviewers. We strongly encourage code deposition in a community repository (e.g. GitHub). See the Nature Portfolio guidelines for submitting code & software for further information.

Data

Policy information about availability of data

All manuscripts must include a data availability statement. This statement should provide the following information, where applicable:

- Accession codes, unique identifiers, or web links for publicly available datasets
- A description of any restrictions on data availability
- For clinical datasets or third party data, please ensure that the statement adheres to our policy

Source data for Figs. 1-6 and Extended Data Figs. 1-8 are provided with this paper; TCGA genomic and clinical data is available through the National Cancer Institute's Genomic Data Commons web portal (https://portal.gdc.cancer.gov/); SILAC MS/MS proteomic mass spectrometry proteomics data generated in this study have been deposited in the ProteomeXchange Consortium via the PRIDE partner repository with the accession number PXD047094 (https://proteomecentral.proteomexchange.org/cgi/GetDataset?ID=PXD047094); bulk RNAseq data generated in this study have been deposited in the Gene Expression Omnibus under accession number GSE164433 (https://www.ncbi.nlm.nih.gov/geo/query/acc.cgi?acc=GSE164433).

Research involving human participants, their data, or biological material

Policy information about studies with human participants or human data. See also policy information about sex, gender (identity/presentation), and sexual orientation and race, ethnicity and racism.

Reporting on sex and gender	N/A
Reporting on race, ethnicity, or other socially relevant groupings	N/A
Population characteristics	N/A
Recruitment	N/A
Ethics oversight	N/A

Note that full information on the approval of the study protocol must also be provided in the manuscript.

Field-specific reporting

Please select the one below that is the best fit for your research. If you are not sure, read the appropriate sections before making your selection.

☒ Life sciences ☐ Behavioural & social sciences ☐ Ecological, evolutionary & environmental sciences

For a reference copy of the document with all sections, see nature.com/documents/nr-reporting-summary-flat.pdf

Life sciences study design

All studies must disclose on these points even when the disclosure is negative.

Sample size	The sample size for all in vitro and in vivo experiments shown in the paper was chosen to obtain statistical power, in conformity to accepted standard sample size in a number of previous publications using the similar approaches.
Data exclusions	No data were excluded from the analyses of this study.
Replication	Reported results considered biological replicates (in vivo n=5-11, and in vitro n= or >3). All details on biological and technical replicates are provided in the corresponding figures and/or figure legends. All the in vitro experiments and data shown in this manuscript have been repeated independently at least three times with similar results as provided.
Randomization	Allocation was random.
Blinding	Blinding was not done as most of the experiments were carried out by one or two persons and the data analyzed by the same person. It was not feasible during the course of the study to have at least 1-2 individuals for each experiment. No data was excluded in this study and all analyses were performed in a quantitative and objective way.

Reporting for specific materials, systems and methods

We require information from authors about some types of materials, experimental systems and methods used in many studies. Here, indicate whether each material, system or method listed is relevant to your study. If you are not sure if a list item applies to your research, read the appropriate section before selecting a response.

Materials & experimental systems

n/a	Involved in the study
☐	☒ Antibodies
☐	☒ Eukaryotic cell lines
☒	☐ Palaeontology and archaeology
☐	☒ Animals and other organisms
☒	☐ Clinical data
☒	☐ Dual use research of concern
☐	☒ Plants

Methods

n/a	Involved in the study
☒	☐ ChIP-seq
☒	☐ Flow cytometry
☒	☐ MRI-based neuroimaging

Antibodies

Antibodies used

Antibodies for immunoprecipitation:
p53 (DO-1)(SILAC -IP)(Santa Cruz, sc-126 AC, 20 ul/1 × 107 cell lysate);
p53 (Pab240)(SILAC -IP)(Santa Cruz, sc-99 AC, 20 ul /1 × 107 cell lysate);
HA (C29F4)(Cell Signaling, 3724, 2-4 ug/mg lysate);
MCM5 (Bethyl, A300-195A, 2-4 ug/mg lysate);
p53 (DO-1)(Santa Cruz, sc-126x, 2-4 ug/mg lysate);
p53 (Pab240)(Santa Cruz, sc-99, 2-4 ug/mg lysate);
His (Millipore, 05-949, 2-4 ug/mg lysate);
Flag (Sigma-Aldrich, F1804, 2-4 ug/mg lysate);

Antibodies for Western blotting:
p53 (DO-1) (Santa Cruz, sc-126, 1:1000);
p53 (1C12) (Cell Signaling, 2524, 1:100);
V5 (SV5-Pk1) (Thermo Fisher, R960-25, 1:1000);
Flag (Sigma-Aldrich, F1804, 1:5000);
HA (Cell Signaling, 3724, 1:1000);
MCM2 (Cell Signaling, 4007S, 1:1000);
MCM3 (Cell Signaling, 4012S, 1:1000);
MCM4 (G-7) (Santa Cruz, sc-28317, 1:1000);
MCM5 (E-10) (Santa Cruz, sc-165994, 1:1000);
MCM5 (Bethyl, A300-195A, 1:5000)
MCM6 (GeneTex, GTX129216, 1:3000);
MCM7 (Cell Signaling, 3735T, 1:1000);
RPA32 (4E4) (Cell Signaling, 2208, 1:1000);
MEK1/2 (D1A5) (Cell Signaling, 8727, 1:5000);
ORC2 (3G6) (Cell Signaling, 4736S, 1:1000);
NFkB-p100/52 (Cell Signaling, 4882);
RelB (C1E4) (Cell Signaling, 4922, 1:5000);
phospho-IRF3 (Ser386) (E7J8G) (Cell signaling, 37829, 1:1000);
cGAS (E5V3W) (Cell signaling, 79978s, 1:1000);
Sting (D2P2F) (Cell Signaling, 136477S, 1:1000);
phospho-NFkB-p65 (Ser 536) (Santa Cruz, sc-101752, 1:1000);
Actin (Sigma-Aldrich, A1978, 1:10000);
Anti-Rat IgG-HRP (Cell Signaling, 7077, 1:3000);
Anti-mouse IgG-HRP (Cell Signaling, 7076, 1:3000);
Anti-Rabbit IgG-HRP (Cell Signaling, 7074, 1:3000)

Antibodies for DNA fiber assay
Rat anti-BrdU antibody [Abcam, clone BU1/75 (ICR1), ab6326, 1:600];
Mouse anti-BrdU antibody (BD Biosciences, clone B44, 347580, 1:300);
Anti-Rat Alexa Fluor 568 (ThermoFisher, A11077, 1:500);
Anti-Mouse Alexa Fluor 488 (ThermoFisher, A32723, 1:500).

Antibodies for immunofluorescence staining
p53 (DO-1) (Santa Cruz, sc-126, 1:100);
ssDNA (Sigma-Aldrich, clone TNT-3, MAB3868, 1:50);
dsDNA (Sigma-Aldrich, clone AE-2, MAB1293, 1:50);
RPA32 (4E4) (Cell Signaling, 2208, 1:50);
RelB (Sigma-Aldrich, HPA040506,1:50);
Rad51 [EPR3040(3)] (Abcam, ab133534,1:50);
Alexa Fluor 647 anti-mouse IgG (ThermoFisher, A21235, 1:2000);
Alexa Fluor 647 anti-rabbit IgG (ThermoFisher, A21244, 1:2000);
Alexa Fluor 488 anti-mouse IgG (ThermoFisher, A11001,1:2000);
Alexa Fluor 594 anti-rat IgG (ThermoFisher, A11007,1:2000).

Antibodies for IHC
p53 (DO-7) (DaKo, M7001, 1:50);
p53 (CM5) (Leica Biosystems, NCL-L-p53-CM5p, 1:100)

RelB (Sigma-Aldrich, HPA040506, 1:100);
RelB (ThermoFisher, PA5-27679, 1:1000)
Sting (D2P2F) (Cell Signaling, 13647, 1:100);
Goat Anti-Rabbit IgG Antibody (H+L), HRP Conjugated (Genesee Scientific, 20-303, 1:5000);
Goat Anti-mouse IgG Antibody (H+L), HRP Conjugated (Genesee Scientific, 20-304, 1:5000).

Antibodies for multiplex IHC
CD8α (D4W2Z) XP® Rabbit mAb (Cell Signaling, 98941, 1:500);
CD3ε (E4T1B) (Cell Signaling, 78588, 1:500);
RelB (ThermoFisher, PA5-27679, 1:1000).

Antibodies for MIBI
Granzyme B (D6E9W) (Ionpath, 715002, 1:100);
CD11c (D1V9Y) (Ionpath, 714402, 1:100);
F4/80 (D2S9R) (Ionpath, 715603, 1:100);
CD11b (EPR1344) (Ionpath, 715504, 1:100);
CD8α (D4W2Z) XP® Rabbit mAb (Cell Signaling, 98941, 1:100).

Validation

Antibodies for immunoprecipitation:
Mouse anti-p53 (DO-1) was validated for immunoprecipitation (IP), immunoblotting (IB), immunofluorescence staining (IF) and immunohistochemistry (IHC) in a human cell line. https://www.scbt.com/p/p53-antibody-do-1
Mouse anti-p53 (Pab240) was validated for IB, IP and IF in a human cell line. https://www.scbt.com/p/p53-antibody-pab-240
Rabbit anti-HA (C29F4) was validated for IB, IP, IHC and IF in a human cell line. https://www.cellsignal.com/products/primary-antibodies/ha-tag-c29f4-rabbit-mab/3724
Rabbit anti-MCM5 was validated for IB, IP, and IHC in a human cell line. https://www.fortislife.com/products/primary-antibodies/rabbit-anti-mcm5-antibody/BETHYL-A300-195
Mouse anti-His was validated for IB, IP and IHC in a human cell line. https://www.sigmaaldrich.com/US/en/product/mm/05949
Mouse anti-Flag was validated for IB, IP, IF and IHC in a human cell line. https://www.sigmaaldrich.com/US/en/product/sigma/f1804

Antibodies for Western blotting:
Mouse anti-p53 (DO-1) was validated for IB, IP, IF and IHC in a human cell line. https://www.scbt.com/p/p53-antibody-do-1
Mouse anti-p53 (1C12) was validated for IB in both a human cell line and a mouse cell line. https://www.cellsignal.com/products/primary-antibodies/p53-1c12-mouse-mab/2524
Mouse anti-V5 (SV5-Pk1) was validated for IB in a human cell line. https://www.thermofisher.com/antibody/product/V5-Tag-Antibody-clone-SV5-Pk1-Monoclonal/R960-25
Mouse anti-Flag was validated for IB, IP, IF and IHC in a human cell line. https://www.sigmaaldrich.com/US/en/product/sigma/f1804
Rabbit anti-HA (C29F4) was validated for IB, IP, IHC and IF in a human cell line. https://www.cellsignal.com/products/primary-antibodies/ha-tag-c29f4-rabbit-mab/3724
Rabbit anti-MCM2 was validated for IB in a human cell line. https://www.cellsignal.com/products/primary-antibodies/mcm2-antibody/4007
Rabbit anti-MCM3 was validated for IB in a human cell line. https://www.cellsignal.com/products/primary-antibodies/mcm3-antibody/4012
Mouse anti-MCM4 was validated for IB in a human cell line.
https://www.scbt.com/p/mcm4-antibody-g-7
Mouse anti-MCM5 was validated for IB in a human cell line.
https://www.scbt.com/p/mcm5-antibody-e-10
Rabbit anti-MCM5 was validated for IB in a human cell line. https://www.fortislife.com/products/primary-antibodies/rabbit-anti-mcm5-antibody/BETHYL-A300-195
Rabbit anti-MCM6 was validated for IB in a human cell line. https://www.genetex.com/Product/Detail/MCM6-antibody/GTX129216
Rabbit anti-MCM7 was validated for IB in a human cell line. https://www.cellsignal.com/products/primary-antibodies/mcm7-d10a11-xp-rabbit-mab/3735
Rat anti-RPA32 (4E4) was validated for IB in a human cell line. https://www.cellsignal.com/products/primary-antibodies/rpa32-rpa2-4e4-rat-mab/2208
Rabbit anti-MEK1/2 (D1A5) was validated for IB in a human cell line.
https://www.cellsignal.com/products/primary-antibodies/mek1-2-d1a5-rabbit-mab/8727
Rabbit anti-ORC2 (3G6) was validated for IB in a human cell line.
https://www.cellsignal.com/products/primary-antibodies/orc2-3g6-rat-mab/4736
Rabbit anti-NFkB-p100/52 was validated for IB in a human cell line. https://www.cellsignal.com/products/primary-antibodies/nf-kb2-p100-p52-antibody/4882
Rabbit anti-RelB was validated for IB in both a human cell line and a mouse cell line. https://www.cellsignal.com/products/primary-antibodies/relb-c1e4-rabbit-mab/4922
Rabbit anti-phospho-IRF3 (Ser386) (E7J8G) was validated for IB in a human cell line. https://www.cellsignal.com/products/primary-antibodies/phospho-irf-3-ser386-e7j8g-xp-rabbit-mab/37829
Rabbit anti-cGAS (E5V3W) was validated for IB in a human cell line. https://www.cellsignal.com/products/primary-antibodies/cgas-e5v3w-rabbit-mab/79978
Rabbit anti-Sting (D2P2F) was validated for IB in a human cell line. https://www.cellsignal.com/products/primary-antibodies/sting-d2p2f-rabbit-mab/13647
Rabbit anti-phospho-NFkB-p65 (Ser 536) was validated for IB in a human cell line.
https://www.scbt.com/p/p-nfkappab-p65-antibody-ser-536-human
Mouse anti-Actin was validated for IB in a human cell line. https://www.sigmaaldrich.com/US/en/product/sigma/a1978
Anti-Rat IgG-HRP was validated for IB in a human cell line. https://www.cellsignal.com/products/secondary-antibodies/anti-rat-igg-hrp-linked-antibody/7077
Anti-Mouse IgG-HRP was validated for IB in a human cell line. https://www.cellsignal.com/products/secondary-antibodies/anti-mouse-igg-hrp-linked-antibody/7076

Anti-Rabbit IgG-HRP was validated for IB in a human cell line.
https://www.cellsignal.com/products/secondary-antibodies/anti-rabbit-igg-hrp-linked-antibody/7074

Antibodies for DNA fiber assay
Rat anti-BrdU antibody was validated for IF in a human cell line. https://www.abcam.com/products/primary-antibodies/brdu-antibody-bu175-icr1-proliferation-marker-ab6326.html
Mouse anti-BrdU antibody was validated for IF in a human cell line. https://www.bdbiosciences.com/en-us/products/reagents/flow-cytometry-reagents/clinical-discovery-research/single-color-antibodies-ruo-gmp/purified-mouse-anti-brdu.347580
Goat anti-Rat IgG Alexa Fluor 568 was validated for IF in a human cell line. https://www.thermofisher.com/antibody/product/Goat-anti-Rat-IgG-H-L-Cross-Adsorbed-Secondary-Antibody-Polyclonal/A-11077
Goat anti-Mouse Alexa Fluor 488 was validated for IF in a human cell line. https://www.thermofisher.com/antibody/product/Goat-anti-Mouse-IgG-H-L-Highly-Cross-Adsorbed-Secondary-Antibody-Polyclonal/A32723

Antibodies for immunofluorescence staining
Mouse anti-p53 (DO-1) was validated for IF in a human cell line. https://www.scbt.com/p/p53-antibody-do-1
Mouse anti-ssDNA was validated for IF in a human cell line. https://www.sigmaaldrich.com/US/en/product/mm/mab3868

Mouse anti-dsDNA was validated for IF in a human cell line. https://www.sigmaaldrich.com/US/en/product/mm/mab1293
Rat anti-RPA32 was validated for IF in a human cell line. https://www.cellsignal.com/products/primary-antibodies/rpa32-rpa2-4e4-rat-mab/2208
Rabbit anti-RelB was validated for IF in a human cell line. https://www.sigmaaldrich.com/US/en/product/sigma/hpa040506
Rabbit anti-Rad51 was validated for IF in a human cell line. https://www.abcam.com/products/primary-antibodies/rad51-antibody-epr40303-ab133534.html
Alexa Fluor 647 Goat anti-mouse IgG was validated for IF in a human cell line. https://www.thermofisher.com/antibody/product/Goat-anti-Mouse-IgG-H-L-Cross-Adsorbed-Secondary-Antibody-Polyclonal/A-21235
Alexa Fluor 647 Goat anti-rabbit IgG was validated for IF in a human cell line. https://www.thermofisher.com/antibody/product/Goat-anti-Rabbit-IgG-H-L-Cross-Adsorbed-Secondary-Antibody-Polyclonal/A-21244
Alexa Fluor 488 Goat anti-mouse IgG was validated for IF in a human cell line. https://www.thermofisher.com/antibody/product/Goat-anti-Mouse-IgG-H-L-Cross-Adsorbed-Secondary-Antibody-Polyclonal/A-11001
Alexa Fluor 594 Goat anti-rat IgG was validated for IF in a human cell line. https://www.thermofisher.com/antibody/product/Goat-anti-Rat-IgG-H-L-Cross-Adsorbed-Secondary-Antibody-Polyclonal/A-11007

Antibodies for IHC
Mouse anti-p53 (DO-7) was validated for IHC in a human cell line. https://www.agilent.com/en/product/immunohistochemistry/antibodies-controls/primary-antibodies/p53-protein-%28concentrate%29-76616
p53 (CM5) was validated for IHC in a mouse cell line. https://shop.leicabiosystems.com/us/ihc-ish/ihc-primary-antibodies/pid-p53-protein-cm5
Rabbit anti-RelB was validated for IHC in a human cell line. https://www.sigmaaldrich.com/US/en/product/sigma/hpa040506
Rabbit anti-RelB was validated for IHC in both a human cell line and a mouse cell line. https://www.thermofisher.com/antibody/product/RelB-Antibody-Polyclonal/PA5-27679
Rabbit anti-Sting (D2P2F) was validated for IHC in both a human cell line and mouse cell line. https://www.cellsignal.com/products/primary-antibodies/sting-d2p2f-rabbit-mab/13647
Goat Anti-Rabbit IgG Antibody (H+L), HRP Conjugated was validated for IHC. https://geneseesci.com/shop-online/product-details/20-303/prometheus-protein-biology-products-20-303-goat-anti-rabbit-igg-h-l-hrp-linked-whole-ab-secondary-antibody-1mg-ml-500ul-unit
Goat Anti-mouse IgG Antibody (H+L), HRP Conjugated was validated for IHC https://geneseesci.com/shop-online/product-details/20-304/prometheus-protein-biology-products-20-304-goat-anti-mouse-igg-h-l-hrp-linked-whole-ab-secondary-antibody-1mg-ml-500ul-unit

Antibodies for multiplex IHC
Rabbit anti-CD8α (D4W2Z) XP® was validated for IHC and IB in a mouse cell. line.https://www.cellsignal.com/products/primary-antibodies/cd8a-d4w2z-xp-rabbit-mab/98941
Rabbit anti-CD3ε (E4T1B) was validated for IHC, IF and IB in a mouse cell. https://www.cellsignal.com/products/primary-antibodies/cd3e-e4t1b-xp-rabbit-mab/78588
Rabbit anti-RelB was validated for IHC in both a human cell line and a mouse cell line. https://www.thermofisher.com/antibody/product/RelB-Antibody-Polyclonal/PA5-27679

Antibodies for MIBI
Rabbit anti-Granzyme B (D6E9W) was validated for IHC in a mouse cell. https://www.ionpath.com/granzyme-b-d6e9w-antibody/
Rabbit anti-CD11c (D1V9Y) was validated for IHC in a mouse cell. https://www.ionpath.com/cd11c-d1v9y-antibody/
Rabbit anti-F4/80 (D2S9R) was validated for IHC in a mouse cell. https://www.ionpath.com/f4-80-d2s9r-antibody-ffpe/
CD11b (EPR1344) was validated for IHC in a mouse cell. https://www.ionpath.com/cd11b-epr1344-antibody/
Rabbit anti-CD8α (D4W2Z) XP® was validated for IHC and IB in a mouse cell. line. https://www.cellsignal.com/products/primary-antibodies/cd8a-d4w2z-xp-rabbit-mab/98941

Eukaryotic cell lines

Policy information about cell lines and Sex and Gender in Research

Cell line source(s): Immortalized human normal head and neck epithelial hTERT HAK cl41 cells (provided by Dr. Aloysius J. Klingelhutz, University of Iowa Research Foundation); HEK293-FT (Thermofisher, R70007); UM-SCC-1 (provided by Dr. Thomas E. Carey, University of Michigan); MDA1586 (provided by Dr. Peter Sacks, New York University); PCI-15B (provided by Dr. Jennifer Grandis, University of Pittsburgh School of Medicine); Detroit 562 (ATCC, CCL-138); HN5 (provided by Dr. D. M. Easty, Ludwig Institute

for Cancer Research); Ca9-22 (Japan Health Science Research Resource Bank); 4T1 (MD Anderson Cytogenetics and Cell Authentication Core).
The following stable cell lines established in our laboratory from above parental cell lines were also used in the current study:
hTERT HAK cl41-KOp53-c1-pBabe
hTERT HAK cl41-KOp53-c1-p53WT
hTERT HAK cl41-KOp53-c1-p53R273H
hTERT HAK cl41-KOp53-c1-p53G245D
hTERT HAK cl41-KOp53-c1-pBabe-PLVX
hTERT HAK cl41-KOp53-c1-pBabe-MCM5
hTERT HAK cl41-KOp53-c1-p53R273H-PLVX
hTERT HAK cl41-KOp53-c1-p53R273H-MCM5
hTERT HAK cl41-KOp53-c1-p53G245D-PLVX
hTERT HAK cl41-KOp53-c1-p53G245D-MCM5
UM-SCC-1-pBabe
UM-SCC-1-p53E336X
UM-SCC-1-p53R273H
UM-SCC-1-p53R248Q
UM-SCC-1-p53G245D
UM-SCC-1-pBabe-PGIPz
UM-SCC-1-pBabe-shRelB1
UM-SCC-1-p53R273H-PGIPz
UM-SCC-1-p53R273H-shcGAS-1
UM-SCC-1-p53R273H-shcGAS-2
UM-SCC-1-p53R273H-shSting-1
UM-SCC-1-p53R273H-shSting-2
UM-SCC-1-p53R273H-shRelB-1
UM-SCC-1-p53R273H-shRelB-2
UM-SCC-1-p53G245D-PGIPz
UM-SCC-1-p53G245D-shSting-1
UM-SCC-1-p53G245D-shRelB-1
UM-SCC-1-p53G245D-shRelB-2
UM-SCC-1-pBabe-PLVX
UM-SCC-1-pBabe-MCM5
UM-SCC-1-p53R273H-PLVX
UM-SCC-1-p53R273H-MCM5
UM-SCC-1-p53R273H-MCM5 R732A/K734A
UM-SCC-1-p53G245D-PLVX
UM-SCC-1-p53G245D-MCM5
UM-SCC-1-pBabe-GFP-luciferase-inducible MCM2
UM-SCC-1-p53R273H-GFP-luciferase-inducible MCM2
UM-SCC-1-pBabe-GFP-luciferase-inducible MCM5
UM-SCC-1-p53R273H-GFP-luciferase-inducible MCM5
MDA1586-lenti
MDA1586-shp53
MDA1586-lenti-shNT-INDU
MDA1586-shp53-shNT-INDU
MDA1586-shp53-shMCM5-C-INDU
MDA1586-shp53-shMCM5-G-INDU
PCI-15B-lenti
PCI-15B-shp53
PCI-15B-lenti-shNT-INDU
PCI-15B-shp53-shNT-INDU
PCI-15B-shp53-shMCM5-C-INDU
PCI-15B-shp53-shMCM5-G-IND
4T1-PLVX-pLKO.1_NT
4T1-R270H-pLKO.1_NT
4T1-R270H-shRelB1
4T1-R270H-shRelB2

Authentication

All the cell lines used in this study including established stable cell lines were authenticated and verified by short tandem repeat (STR) profiling performed on cellular DNA submitted to the MD Anderson Cytogenetics and Cell Authentication Core Facility or the Fragment Analysis Facility of Johns Hopkins University. The detailed STR allelic patterns of each cell line are listed in Supplementary Data 2.

Mycoplasma contamination

We routinely treated cell lines with PlasmocureTM (50 ug/ml) for 2 weeks before experiments, which is usually sufficient to completely eliminate mycoplasma (see PlasmocureTM information, InvivoGen, anti-pc). We did not conduct routine test for mycoplasma contamination.

Commonly misidentified lines
(See ICLAC register)

NO

Animals and other research organisms

Policy information about studies involving animals; ARRIVE guidelines recommended for reporting animal research, and Sex and Gender in Research

Laboratory animals	5- to 6-week-old athymic male nude mice (Harlan, athymic nu/nu); 8-week-old female BALB/cJ and NOD SCID (NOD.Cg-Prkdcscid/J) mice (Jackson Laboratories).
Wild animals	NO
Reporting on sex	For animal studies of human oral cancer UM-SCC-1 and MDA1586 stable cell lines, male nude mice were used. For animal studies of 4T1 stable cell line, female mice were used. Since men with smoking histories have high incidence of oral cancer, we selected male nude mice for human cell lines' studies. 4T1 cell is breast cancer cell line, female BALB/c and SCID mice were used for its study.
Field-collected samples	NO
Ethics oversight	Animal experiments were performed in accordance with protocols approved by the MD Anderson Cancer Center Institutional Animal Care and Use Committee (IACUC).

Note that full information on the approval of the study protocol must also be provided in the manuscript.

Dual use research of concern

Policy information about dual use research of concern

Hazards

Could the accidental, deliberate or reckless misuse of agents or technologies generated in the work, or the application of information presented in the manuscript, pose a threat to:

No	Yes	
☒	☐	Public health
☒	☐	National security
☒	☐	Crops and/or livestock
☒	☐	Ecosystems
☒	☐	Any other significant area

Experiments of concern

Does the work involve any of these experiments of concern:

No	Yes	
☒	☐	Demonstrate how to render a vaccine ineffective
☒	☐	Confer resistance to therapeutically useful antibiotics or antiviral agents
☒	☐	Enhance the virulence of a pathogen or render a nonpathogen virulent
☒	☐	Increase transmissibility of a pathogen
☒	☐	Alter the host range of a pathogen
☒	☐	Enable evasion of diagnostic/detection modalities
☒	☐	Enable the weaponization of a biological agent or toxin
☒	☐	Any other potentially harmful combination of experiments and agents

Plants

Seed stocks	NO and N/A
Novel plant genotypes	NO and N/A
Authentication	NO and N/A
